# Raman spectroscopic insight into osteoarthritic cartilage regeneration by mRNA therapeutics encoding cartilage-anabolic transcription factor Runx1

**DOI:** 10.1016/j.mtbio.2022.100210

**Published:** 2022-01-29

**Authors:** Giuseppe Pezzotti, Wenliang Zhu, Yuki Terai, Elia Marin, Francesco Boschetto, Komei Kawamoto, Keiji Itaka

**Affiliations:** a Ceramic Physics Laboratory, Kyoto Institute of Technology, Sakyo-ku, Matsugasaki, 606-8585, Kyoto, Japan; b Department of Biofunction Research, Institute of Biomaterials and Bioengineering, Tokyo Medical and Dental University (TMDU), 2-3-10 Kanda-Surugadai, Chiyoda-ku, Tokyo, 101-0062, Japan; c Department of Periodontology, School of Dental Medicine, Tsurumi University, 2-1-3, Tsurumi, Tsurumi-ku, Yokohama, Japan

**Keywords:** Cartilage regeneration, mRNA therapeutics, Cartilage-anabolic transcription factor, Raman spectroscopy

## Abstract

While joint arthroplasty remains nowadays the most popular option available to repair chronically degenerated osteoarthritic joints, possibilities are recently emerging for regeneration of damaged cartilage rather than its replacement with artificial biomaterials. This latter strategy could allow avoiding the quite intrusive surgical procedures associated with total joint replacement. Building upon this notion, we first apply Raman spectroscopy to characterize diseased cartilage in a mice model of instability-induced knee osteoarthritis (OA) upon medial collateral ligament (MCL) and medial meniscus (MM) transections. Then, we examine the same OA model after cartilage regeneration by means of messenger RNA (mRNA) delivery of a cartilage-anabolic runt-related transcription factor 1 (RUNX1). Raman spectroscopy is shown to substantiate at the molecular scale the therapeutic effect of the *Runx1* mRNA cartilage regeneration approach. This study demonstrates how the Raman spectroscopic method could support and accelerate the development of new therapies for cartilage diseases.

## Graphical abstract

**Figure:**
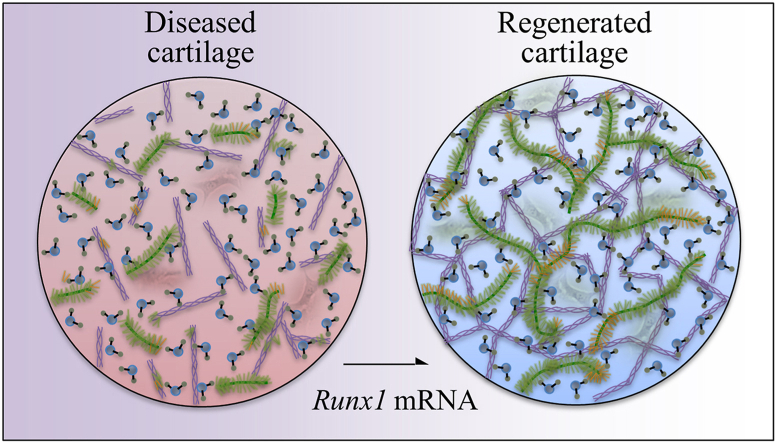

## 1 Introduction

Since being hydrated but avascular, adult human articular cartilage possesses very limited self-repairing ability [1,2]. Consequent to such deficiency in self-healing, cartilage is irreversibly lost in chronic degenerative joint diseases such as osteoarthritis (OA), which imbalances its synthesis and accelerates its degradation [3]. Few exceptions exist to such a general notion. In some traumatic damages reaching the subchondral bone, some intrinsic repair response might be triggered with generation of a fibro-cartilaginous repair tissue. However, such tissue was shown to be poor substitute for hyaline articular cartilage [4,5]. Presently available arthroscopic repair strategies aim at stimulating the intrinsic repair response of cartilage while preserving its functional and mechanical properties. Such approaches include autologous osteochondral transfer, namely, a direct excision of healthy cartilage portions from non-load-bearing regions and their surgical transfer into damaged regions [[6], [7], [8]], transplantation of autologous chondrocytes from healthy to defective cartilage regions [9,10], and, use of mesenchymal stem cells (eventually in combination with biodegradable scaffolds) as tissue source [11]. The regenerative method of autologous osteochondral transfer can provide symptomatic relief, but it only partly improves joint functions. Chondrocyte transplantation in humans (reviewed by Brittberg [12]) gave satisfactory results in 80% of patients after follow-up of 2–9 years [13]. While mesenchymal stem cells, which possess the ability to intrinsically regenerate cartilage and to differentiate into a variety of other connective tissues [14], should first be isolated and proliferated in vitro before being delivered *via* carrier scaffolds in conjunction with specific growth factors to facilitate differentiation and maturation. With human clinical trials ongoing, the mesenchymal cell approach to cartilage tissue repair appears a promising one. In a scenario in which surgical treatment techniques cannot regenerate articular cartilage, regenerative medicine involving stem cells has certainly entered a new stage and is considered a promising way to regenerate articular cartilage [15]. However, stem cell transplantation is yet related to risks of tumorigenesis, immune rejection, and disease transmission, besides introducing functional cell heterogeneity from different individuals [[16], [17], [18], [19]].

In a recent study, we reported about a new disease-modifying strategy for cartilage regeneration based on *in situ* mRNA delivery of a therapeutic, cartilage-anabolic transcription factor, runt-related transcription factor 1 (Runx1) [20]. It is demonstrated that *Runx1* mRNA induced an improved efficacy in the *in vivo* regeneration of mice articular cartilage by intra-articular administration of the mRNA using our original polymer-based carrier, polyplex nanomicelles [[21], [22], [23], [24], [25], [26]]. Owing to the capacity of nanomicelles for smooth tissue penetration, the mRNA could be uniformly introduced into the chondrocytes in the superficial and middle zones of the articular cartilage. Although this innovative therapeutic strategy appears to be quite promising as a new therapeutic approach to osteoarthritic degenerative diseases, it yet remains conspicuously unexplored at the molecular scale.

In this paper, we applied Raman spectroscopy to assess the effectiveness of the *Runx1* mRNA approach to cartilage regeneration. This study builds upon our previous researches of osteoarthritic human cartilage by which we developed and validated a quantitative Raman algorithm for assessing OA progression [27]. Although there are approaches to precisely diagnose the cartilage using laser [28], Raman spectroscopy is a label-free technique, which is capable of both analyzing tissues at the molecular scale and locating degenerative phenomena in human explanted cartilage or in animal models of surgically induced OA, as well as examining collagen denaturation and conformational state change of polysaccharide chains [29]. The Raman method enables to detect subtle changes in the molecular structure that may precede cartilaginous changes in the osteoarthritic joint. Once implemented in the clinical practice, it could become a useful diagnostic tool. We use and expand here previously developed Raman algorithm aiming at quantifying the pathology of articular cartilage in a mice knee OA model and to substantiate the curative effects after *Runx1* mRNA treatment in a time-dependent fashion, in comparison with healthy cartilage obtained from the same mice as control samples. The demonstration of a curative effect by Raman spectroscopy substantiated the regenerative *Runx1* mRNA approach at the molecular scale, while also confirming the usefulness of Raman spectroscopy as a tool in OA diagnostics.

## 2 Experimental procedures

### 2.1 Preparation of specimen of mouse knee cartilage

All the animal experimental protocols were approved by the Institutional Animal Care and Use Committee of Tokyo Medical and Dental University (TMDU), and all experiments were performed in accordance with relevant guidelines and regulations. The right knee joints of 8-week-old Balb/c mice (purchased from Sankyo Labo Service Corp., Tokyo, Japan) were used for preparing specimen of cartilage. A mouse knee OA model, in which the OA changes was induced by joint instability, was used in this study [30]. Briefly, under general anesthesia, the medial collateral ligament (MCL) was transected, and the medial meniscus (MM) was removed using a surgical microscope. In this model, the cartilage undergoes degeneration with time, and the histologic appearance is visible 8 weeks after the surgery [30].

For Raman spectroscopy, the knee joint was carefully disarticulated so as not to damage the cartilage after euthanasia, and the medial cartilaginous articular surfaces of the tibia were served for the spectroscopy. The specimens were obtained from one to four weeks after the surgery of resecting MCL and MM, and that of receiving *Runx1* mRNA was obtained one week after the mRNA injection (totally 5 weeks after the surgery) as described in the following section. The contralateral intact knee was used as a control. The sample size was around 1 ​cm in length, and the number of specimen for each type of mouse knee is N ​= ​5 for control healthy tibia cartilage, N ​= ​4 for knee joints at 2 and 4 weeks after MCL and MM transection, and N ​= ​3 for the one treated by Runx1 mRNA.

### 2.2 Intra-articular injection of Runx1 mRNA

The intra-articular injection of *Runx1* mRNA was done as previously reported [20]. Briefly, *Runx1* mRNA was prepared by in vitro transcription (IVT) from a DNA template, which was prepared from a pSP73 vector (Promega, Madison, WI, USA) after cloned with a human *Runx1* ORF sequence ([NM_001754.5]; 1443 bps). The IVT was performed using a mMESSAGE mMACHINE T7 ULTRA Kit (Ambion, Invitrogen, Carlsbad, CA, USA), followed by purification using an RNeasy mini kit (QIAGEN, Hilden, Germany) and spectroscopic measurement of concentration at 260 ​nm. Purity of mRNA was assessed spectroscopically based on ultraviolet (UV) absorption, by confirming that ratio of absorbance at 260 and 280 ​nm (A260/A280) was 1.8–2.1, and size of mRNA was evaluated by on-chip capillary electrophoresis using Bioanalyzer Agilent2100 (Agilent, Santa Clara, CA, USA) (Supplementary Fig. 1).

The mRNA was injected into the joint using a polymer-based carrier, polyplex nanomicelle. A block copolymer, polyethylene glycol-polyamino acid (Poly{N-[*N*′-(2-aminoethyl)-2-aminoethyl]aspartamide}) block copolymer (PEG-PAsp(DET)) was synthesized as previously described [31]. The molecular weight of PEG was 43 ​k, and the polymerization degree of PAsp(DET) was 63, determined by using 1H NMR measurement (JEOL EX300 spectrometer, JEOL, Tokyo, Japan). The polyplex nanomicelle was formed following a mixing procedure of the mRNA and block copolymer solutions in 10 ​mM HEPES buffer (pH 7.3), by adjusting the N/P ratio (the residual molar ratio of the polycations amino groups to the mRNA phosphate groups) to be 8. The nanomicelles were revealed to have the size around 50 ​nm with almost neutral surface charge [20]. The final mRNA concentration was fixed to 50 ​μg/mL. For intra-articular injection in the mouse knee, 20 ​μL of polyplex nanomicelle solution containing 1 ​μg mRNA was injected through the patellar tendon using a 30 ​G syringe under anesthesia [32].

### 2.3 Micro-CT imaging

Micro-CT imaging of the knee joints was performed at 4 weeks after the surgery, using the microfocus X-ray computed tomography system CosmoScan FX (Rigaku, Tokyo, Japan) under the following conditions: tube voltage, 90 ​kV and tube current, 110 ​mA. The images were reconstituted to frontal and sagittal planes to identify the joint space.

### 2.4 Histological analysis

Four weeks after the OA surgery, the mice were euthanized and subjected to perfusion fixation with 4% paraformaldehyde [PFA] solution (room temperature) for 24 ​h. Then, the frozen sections were prepared following the procedures reported previously [33]. Briefly, the specimens were completely frozen with hexane dry ice. Next, the frozen specimens were placed in an embedding medium (SCEM), moved into hexane dry ice and completely frozen. The frozen specimens were fixed to a cryomicrotome (CM3050S, Leica Biosystems) maintained at −20 ​°C to the cryochamber and −25 ​°C to the sample holder chuck. Then, the specimens were trimmed with a disposable tungsten carbide blade. An adhesive film (Cryofilm type 3C (16UF), SECTION-LAB Co. Ltd., Japan) was applied onto the cut surface and then frozen sections of 3–5-μm thickness were made at a slow cutting speed to limit damages.

The frozen sections made with the Cryofilm were taken out of the cryochamber and thawed. After 10 ​s, they were immersed in 100% ethanol for 10 ​s. Then, they were placed in 4% PFA for 2 ​min and rinsed in running water for 4 ​min. For the H&E and staining, the sections were stained with Carrazzi's Hematoxylin Solution for 90 ​s and rinsed for 4 ​min. Next, the sections were stained with 0.2% eosin dissolved in distilled water for 10 ​s. For the Toluidine Blue staining, the sections were stained with 0.05% Toluidine Blue Solution (pH 4.1) and rinsed for 30 ​s. After the staining, the sections were rinsed in 100% ethanol, mounted between the Cryofilm and slide glass with a water-soluble mounting medium (SCMM-R2, SECTION-LAB Co. Ltd. Japan), and then the SCMM-R2 was polymerized using ultraviolet light.

### 2.5 Raman spectroscopy

The spectroscopic assessments performed in this study were made by means of a highly spectrally resolved Raman microprobe spectrometer (T-64000, Horiba/Jobin-Yvon, Kyoto, Japan) operating in back-scattering geometry. The excitation source was a 532 ​nm diode laser (SOC Juno, Showa Optronics Co., Ltd, Tokyo, Japan) yielding a power of approximately 10 ​mW at the cartilage surface. The confocal configuration of the laser probe adopted throughout the present experiments corresponded to a 100 ​× ​objective lens, with the pinhole diameter of the cross slit fixed at 100 ​μm. The laser penetration depth achieved was calibrated to be in the order of ∼10 ​μm. The recorded (non-polarized) Raman spectra were averaged over three successive measurements at each selected location with an accumulation time of 60 ​s for one spectral window, and 5 different locations from each knee cartilage were measured by the Raman analyses. A spectral resolution better than 0.15 ​cm^−1^ could be achieved by means of an 1800 lines/mm grating and by systematically recording a selected signal from a neon lamp for the purpose of an internal calibration of the spectrometer. Measurements were performed in controlled ambient temperature of 22 ​°C. Background subtractions and spectral deconvolutions were performed by means of the automatic-fitting algorithms enclosed in a commercially available computational package (LabSpec 5, Horiba/Jobin-Yvon, Kyoto, Japan). In the spectral deconvolution, we used mixed Gaussian/Lorentzian curves to fit spectral sub-bands as follows:

$$I\left(\omega \right)=A\left(g\times \frac{1}{\sqrt{2\pi }w}\text{Exp}\left[-\frac{{\left(\omega -\omega_{p}\right)}^{2}}{2w^{2}}\right]+\left(1-g\right)\times \frac{1}{{\left(\omega -\omega_{p}\right)}^{2}+w^{2}}\right)$$

where ω_p_, *w* and *A* are the peak position, width and area, respectively, and *g* is a parameter showing the fraction of Gauss function (*g* ​= ​0.5 in this study).

The relationship between the observed Raman bands and the vibrational modes of the cartilage molecular structure has been documented and discussed in previous studies [27,34].

### 2.6 Statistical analyses

A one-way analysis of variance (ANOVA) was performed for comparison of biochemical changes detected in Raman fingerprint bands for: (i) fractions of random coil vs. α-helix secondary structures calculated from Amide III ratio of bands located at ∼1240 ​cm^−1^ and ∼1270 ​cm^−1^, respectively; and, (ii) ratios of Raman intensity for the main peaks of chondroitin sulfate and hyaluronic acid at 1063 and 1126 ​cm^−1^, respectively. Differences were considered significant at the *p* ​< ​0.05 level. An unpaired two-sided Student's *t*-test was also applied in order to compare changes between the same parameters within each control group. Also in these latter tests, differences were considered significant at *p* ​< ​0.05.

## 3 Results

### 3.1 Raman spectra of healthy and degenerated cartilage

**Fig. 1:**
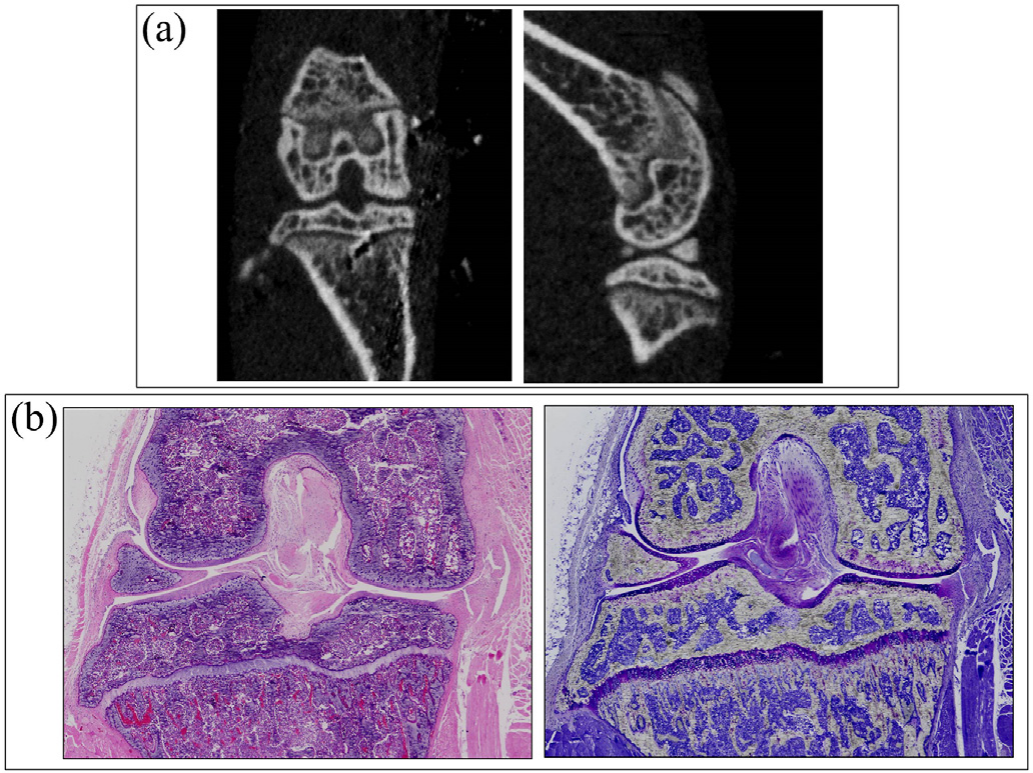
Micro-CT and histological-section images of mice knee 4 weeks after MCL and MM transection. (a) Reconstituted images of sagittal and frontal planes from the raw micro-CT images. (b) Histological sections of the identical knee joint, stained with hematoxylin and eosin (left) or toluidine blue (right).

**Fig. 2:**
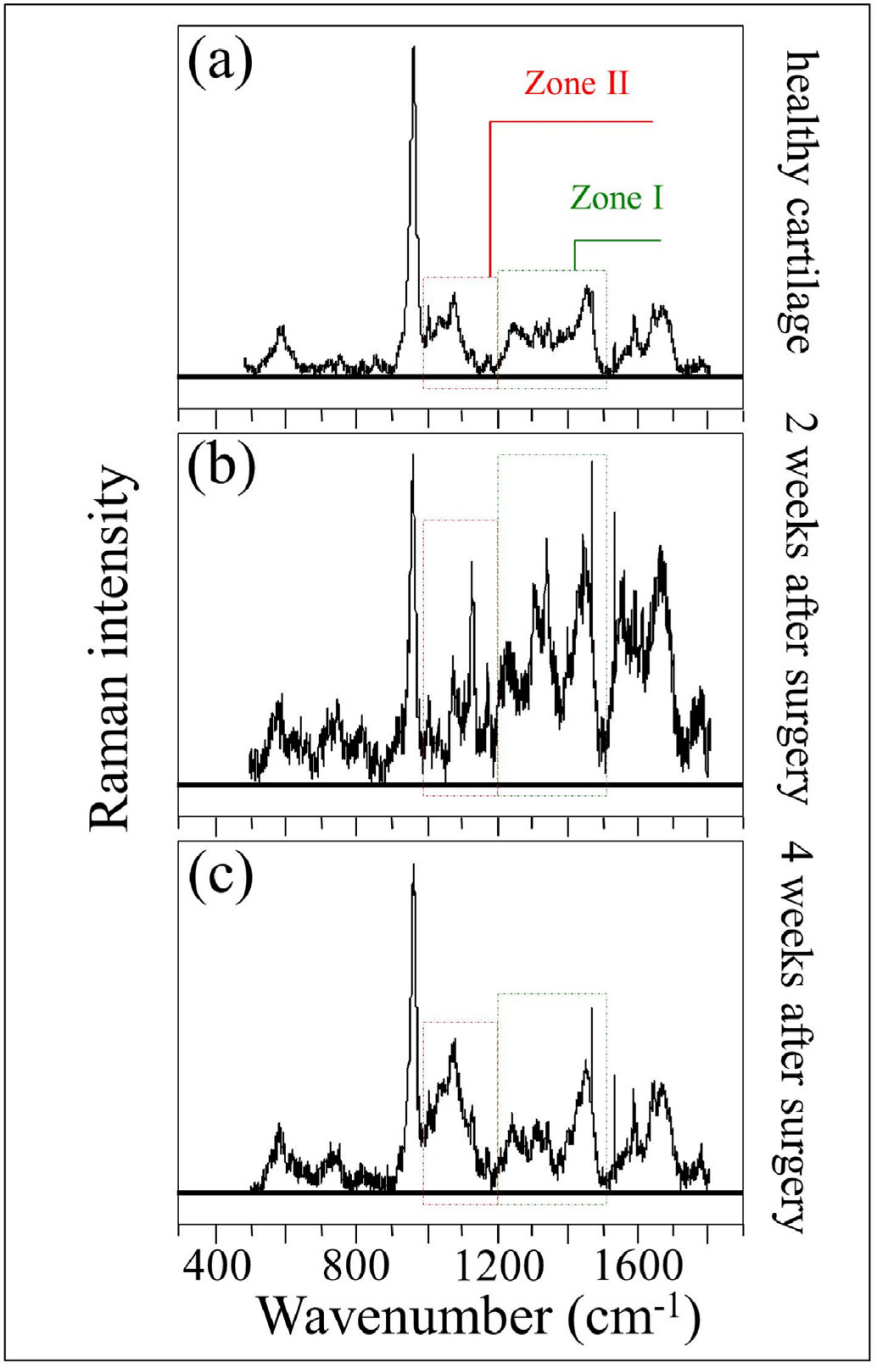
Representative Raman spectra recorded *ex vivo* on (a) control healthy tibia cartilage, and those from knee joints at (b) 2 and (c) 4 weeks after MCL and MM transection. The main regions studied in this paper are emphasized with square insets (cf. labels).

As described in Section 2, in the knee OA model by transecting MCL and MM, the cartilage degeneration becomes histologically evident 8 weeks after the surgery [34]. In this study, we analyzed the cartilage by Raman spectroscopy 2 and 4 weeks after the surgery, when the degenerative changes in the cartilage and the periarticular structures would be barely observed. Indeed, in micro-CT images at 4 weeks (Fig. 1(a)) and 2, 4, and 8 weeks after MCL and MM transection (Supplementary Fig. 2), and the histological sections (Fig. 1(b) and Supplementary Fig. 3) of the knee joints 4 weeks after the surgery, although MM was completely resected in the histological sections, there are almost no significant changes in the cartilage.

For Raman spectroscopy, the medical cartilage of the tibia was exposed by knee disarticulation, and served for the spectroscopy. As shown in Fig. 1, the cartilage spectra showed significant morphological differences between pristine and diseased states, and between diseased states as a function of post-surgery time. Detailed spectral analyses were focused on two specific intervals: Zone I at 1170–1500 ​cm^−1^ and Zone II at 1000–1200 ​cm^−1^ (cf. square insets and labels in Fig. 2). The Amide III region of the spectrum provides structural information on collagen structure.

**Fig. 3:**
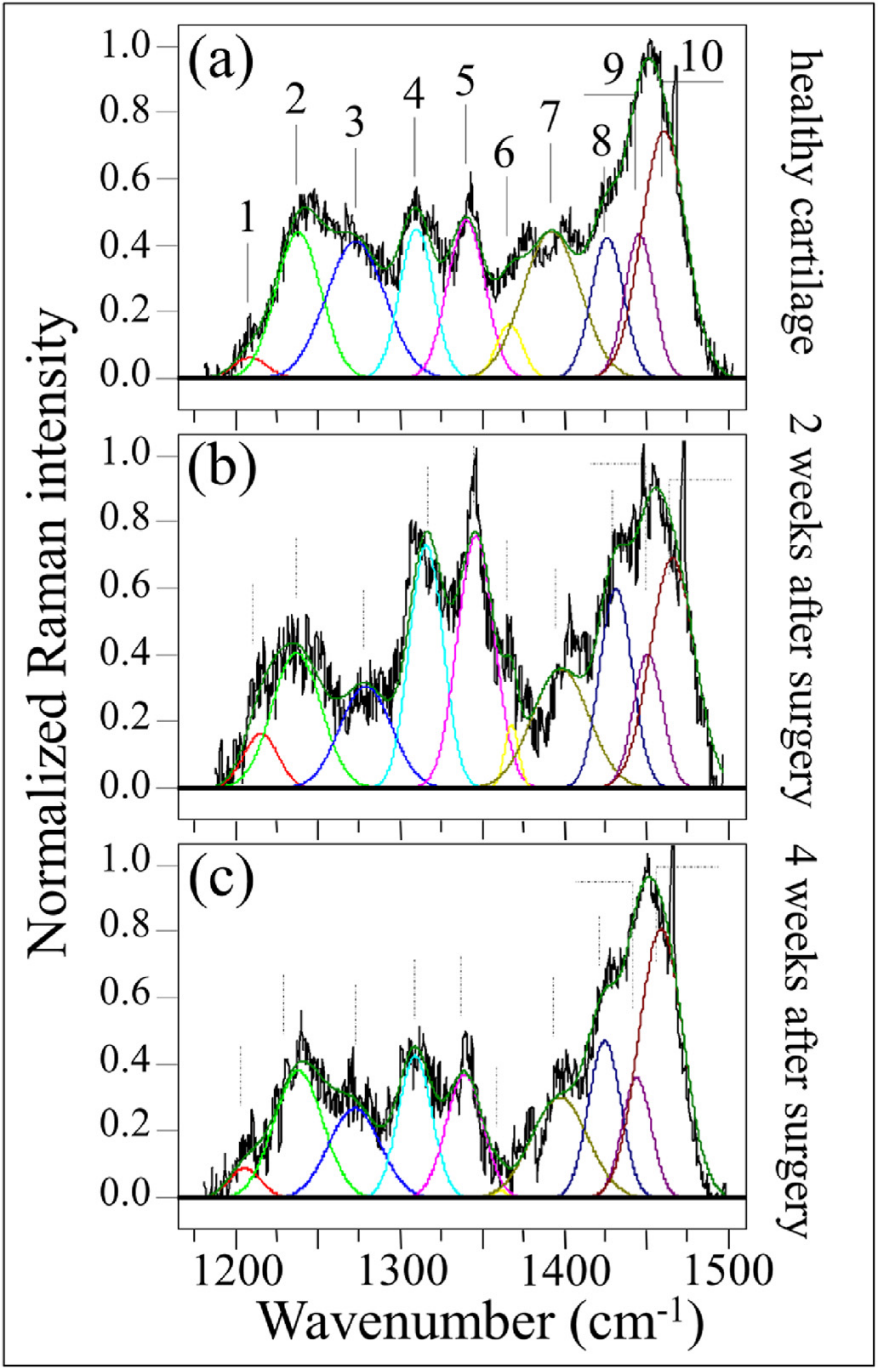
Raman analysis in the spectral zone at 1170–1500 ​cm^−1^: (a) control healthy tibia cartilage, and those from knee joints at (b) 2 and (c) 4 weeks after MCL and MM transection. Deconvolution singled out 10 sub-bands whose frequencies and vibrational assignments are given in Table 1.

**Fig. 4:**
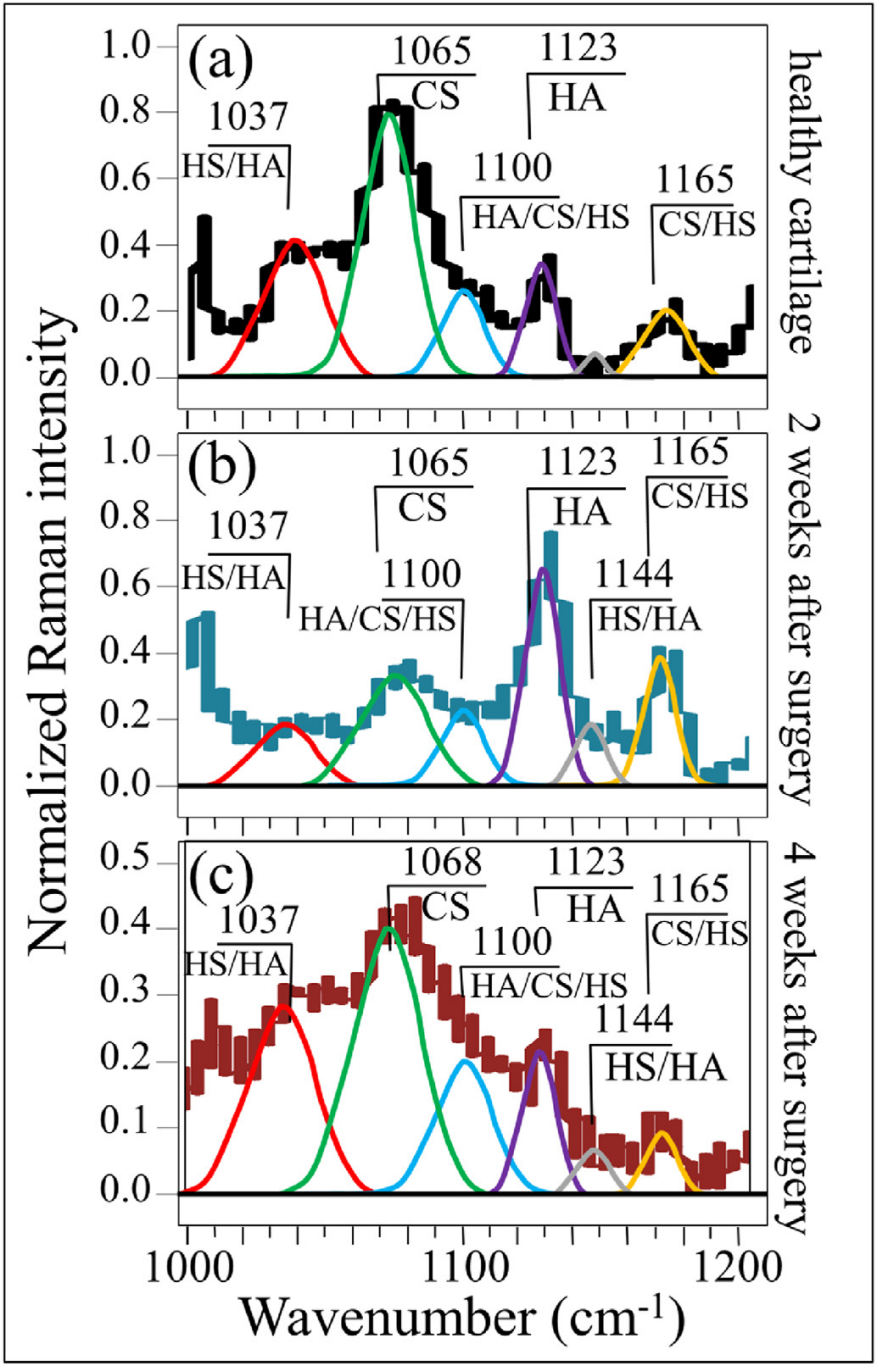
Raman analysis in the spectral zone at 1000–1200 ​cm^−1^: (a) control healthy tibia cartilage, and those from knee joints at (b) 2 and (c) 4 weeks after MCL and MM transection. Spectral frequencies are listed in Table 2 and assignments to specific molecules are given in inset.

These two zones are represented in Fig. 3, Fig. 4, respectively, as collected under healthy and diseased conditions (2 and 4 weeks after the surgery; cf. labels). As the spectra clearly exhibit the presence of different peaks, resolvable from the spectral morphology, Zone I was deconvoluted into 10 sub-bands, which were located with respect to their frequencies and assigned to the respective vibrational origins as shown in Table 1. As seen, significant differences in spectral morphology could be found by comparing spectra from normal knee joint (Fig. 3(a)) and diseased cartilage of the ACL-transected knee joints at 2 and 4 weeks after the surgery (Fig. 3(b) and (c), respectively). A band located at around 1205 ​cm^−1^ can be observed, assigned to the stretching mode of C–C_6_H_5_ in tyrosine and phenylalanine [35]. One important feature is the observed alteration in relative intensity ratio between the Amide III bands at 1240 ​cm^−1^ and 1271 ​cm^−1^ (i.e., the signals labeled as Bands 2 and 3, respectively, in Fig. 3(a)). These bands have the same physical origin (N─H in-plane bending), but they represent different protein secondary structures, namely, random coil disordered phase and ordered α-helix, respectively [36]. The strongest signal in this region appears centered at ∼1451 ​cm^−1^, but it is a composite signal of two sub-bands that can be assigned to methyl CH_3_ and methylene CH_2_ deformation vibrations [37] (i.e., the signals labeled as Bands 9 and 10, respectively, in Fig. 3(a)). Similar to the N─H groups at 1240 ​cm^−1^, the cumulative Raman signal of CH_3_ and CH_2_ groups does not show orientation dependence in polarized probe assessments, but exploit a homogenous Raman scattering [38]. According to this notion, we normalized the spectra to the maximum at around 1451 ​cm^−1^ and attributed the relative increase of the 1240 ​cm^−1^ band after the surgery to an increase in volume fraction of the disordered random coil phase (cf. Fig. 3(b) and (c)). This point will be discussed in more details in the forthcoming Section 4.1.

**Table 1:** Frequencies and vibrational assignments for the deconvoluted Voigtian sub-bands in the Amide III region between 1170 and 1500 ​cm^−1^; the labels refer to those shown in Fig. 3, Fig. 6.

Label	Frequency (cm^−1^)	Physical origin	References
1	1205	C–C_6_H_5_ stretching	[35]
2	1240	Amide III (random coil)	[36]
3	1270	Amide III (α-helix)	[36]
4	1312	Hydrated amide C=O and *N*–H (α-helix)	[39]
5	1340	Hydrated amide C=O and *N*–H (α-helix)	[39]
6	1365	Ring and C–N stretching	[35]
7	1391	CH_2_ deformation	[35]
8	1429	CH_2_ bending	[35]
9	1446	Methyl CH_3_ deformation	[37]
10	1459	Methylene CH_2_ deformation	[37]

Another striking difference in Zone I was the increase of signals at ∼1312 and ∼1340 ​cm^−1^ in the cartilage sample monitored 2 weeks after the surgery (cf. Bands 4 and 5 in Fig. 3(b)). Both these signals belong to α-helix, but were assigned to its hydrated/relaxed structure [39]. Since the hydrogen bonding of water with the C=O group boldly affects the morphology of Amide III scatter, it can be taken as a fingerprint for different physiological states of cartilage in health and disease, as discussed later in Section 4.1.

Bold differences between healthy and diseased cartilage could also be found in Zone II at 1000–1200 ​cm^−1^ (cf. Fig. 4(a), (b), and (c)). In this zone, spectral deconvolution located six distinct sub-bands, which belong to chondroitin sulfate (CS), hyaluronic acid (HA), and heparan sulfate (HS) (cf. labels in Fig. 4 and Table 2) [40]. The peak at 1065 ​cm^−1^, assigned to OSO_3_^−^ symmetric stretching [40], is a fingerprint of CS, since HA does not contain sulfate groups. Conversely, the peak at 1123 ​cm^−1^, which is related to bending vibrations of C–OH and C–H groups [41], is only characteristic of HA. Two additional bands at 1037 and 1100 ​cm^−1^ in this spectral area are contributed with overlapping C–C and C–O stretching vibrations, and C–OH bending vibrations of acetyl groups, respectively [41,42]. These signals are cumulative from CS, HA, and HS, and are hardly suitable as fingerprints for specific compounds. The band at 1165 ​cm^−1^ also contains composite contributions since it arises from sugar ring moieties in glycosaminoglycans and proteoglycans; more specifically, it reflects vibrations of exocyclic (–C–O–C–) inter-molecular groups [43].

**Table 2:** Frequencies and vibrational assignments for the deconvoluted Voigtian sub-bands in the zone at 1000–1200 ​cm^−1^; the labels refer to those shown in Fig. 4, Fig. 6.

Label	Frequency (cm^−1^)	Physical origin	References
1	1037	HS/HA	[41]
2	1065	CS	[40]
3	1100	HA/CS/HS	[42]
4	1123	HA	[44]
5	1144	HS/HA	[41]
6	1168	CS/HS	[43]

The most striking item in the comparison of Raman spectra taken on healthy and diseased cartilage resides in the variation of relative intensity between the CS peak at 1065 ​cm^−1^ and the HA peak at 1123 ​cm^−1^. The former band is stronger than the latter in healthy cartilage (cf. Fig. 4(a)); the opposite trend is observed in diseased cartilage at 2 weeks after the surgery (cf. Fig. 4(b)), while the original trend appears to be restored after 4 weeks in disease (cf. Fig. 4(c))), although relative intensity in the overall glycosaminoglycans zone is reduced by about a half. Kamilari et al. [44] have used the ratio of peak amplitudes of the deconvoluted bands at 1065 and 1123 ​cm^−1^, fingerprints of CS and HA, respectively, to construct a calibration curve that enables quantification of glycosaminoglycans by Raman spectroscopy. According to those calibrations, the content of CS in healthy cartilage was ∼2 times that of HA, while it turned to be ∼1/3 ​at 2 weeks since the surgery, and then restored back to ∼2 times at 4 weeks. The intensity of the band at 1165 ​cm^−1^ increased considerably in the spectrum of diseased cartilage at 2 weeks and then significantly decreased at 4 weeks after the surgery.

This effect can be partly interpreted by enhanced transition of HA from the joint fluid to the cartilage matrix, in consequence of the “loosening” the internal structure of the α-helix configuration described in the past paragraph. In addition, the increased hydration level in the α-helix structure might augment the retention of HA molecules in the cartilage matrix due to the hydrophilic nature of HA. Further, the high rate of glycosylation that takes place during osteoarthrosis development would also be involved [43]. This point will be further discussed in a later section. Urita et al. [45] have reported that alterations in high-mannose type *N*-glycans occurred in both human osteoarthritic cartilage and degraded mouse cartilage. Those authors also analyzed the link between altered *N*-glycan patterns and the mechanisms of cartilage degradation. This point will be discussed in the forthcoming Section 4.1 together with the altered balance between CS and HA.

### 3.2 Raman spectra after treatment by Runx1 mRNA

**Fig. 5:**
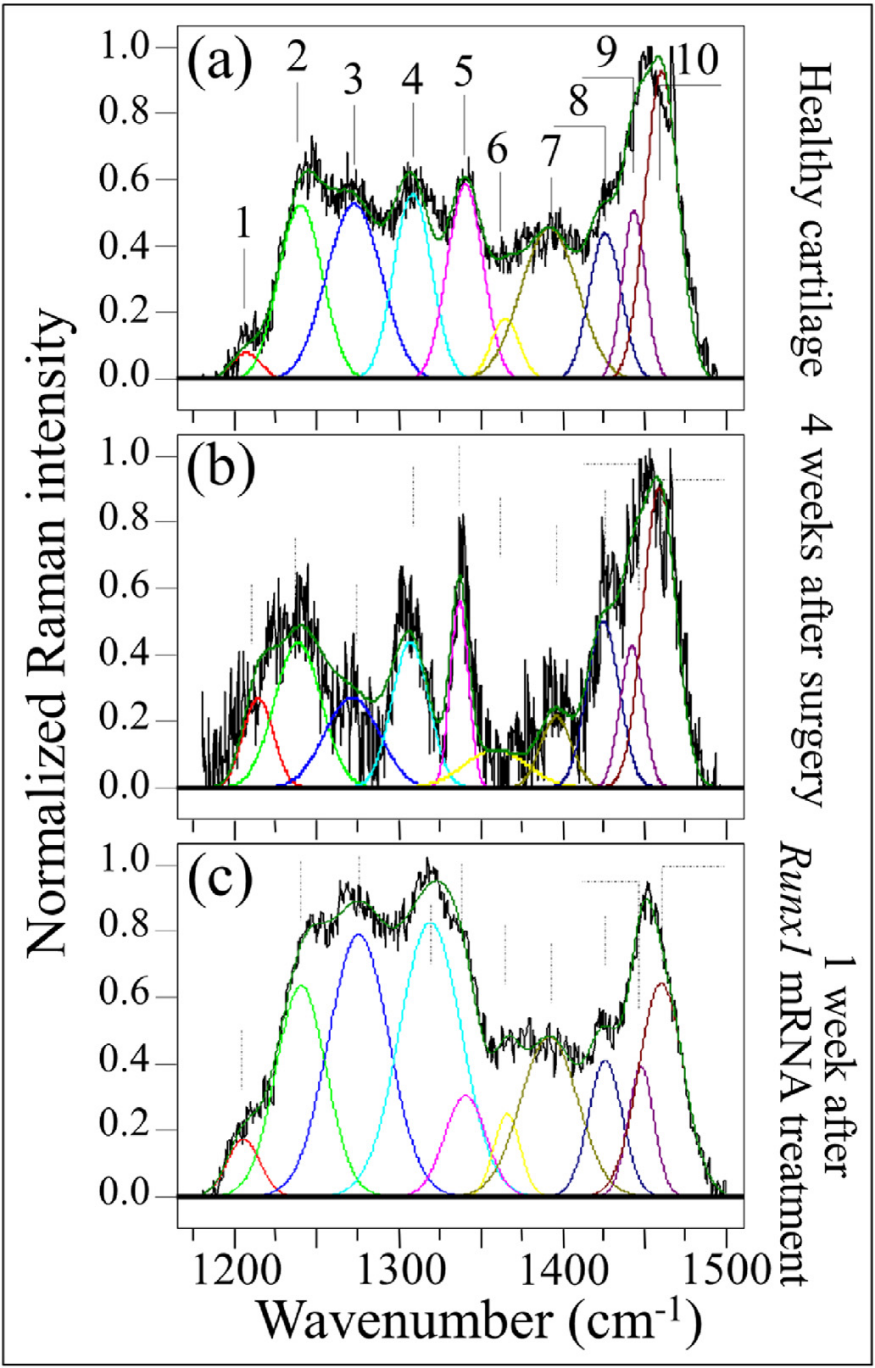
Raman analysis in the spectral zone at 1170–1500 ​cm^−1^: (a) control healthy tibia cartilage, (b) diseased cartilage after 4 weeks since MCL and MM transection, and (c) regenerated cartilage after 4 weeks after MCL and MM transection and one successive week after *Runx1* mRNA administration (deconvolution into 10 sub-bands with frequencies and vibrational assignments given in Table 1).

Fig. 5(a), (b), and (c) give representative (deconvoluted) spectra in Zone I at 1170–1500 ​cm^−1^ for healthy tibia cartilage, for cartilage from the tibia 4 weeks since the surgery, and for cartilage from the tibia after 4 weeks since surgery plus an additional week after *Runx1* mRNA treatment, respectively. Focusing first on the relative intensity ratio between the N─H in-plane bending Amide III bands at 1240 ​cm^−1^ and 1271 ​cm^−1^ (i.e., Bands 2 and 3, respectively), one could note a clear increase in the signal of the disordered phase (Band 2) above the one of the α-helix (Band 3) (cf. Fig. 5(b)), which confirms the trend shown in Fig. 3. However, an additionally important output was the observation of a α-helix signal restored to a value clearly higher than that of the disordered phase 1 week after *Runx1* mRNA treatment (cf. Fig. 5(c)). Moreover, the intensity of the α-helix Band 3 became as strong as that of the signal centered at ∼1451 ​cm^−1^, cumulative from methyl and methylene deformations, to which this spectral zone was originally normalized. The intensity of Band 4 ​at 1312 ​cm^−1^ was comparable with that of Band 3, but the other signal from hydrated α-helix at ∼1340 ​cm^−1^ (Band 5) was clearly lower than that recorded in healthy cartilage. This suggests that the *Runx1* mRNA treatment had a strong effect on the secondary structure of collagen proteins and on their state of hydration as well. Average Raman spectra from each type of cartilage sample will be discussed in the forthcoming Section 4.1.

**Fig. 6:**
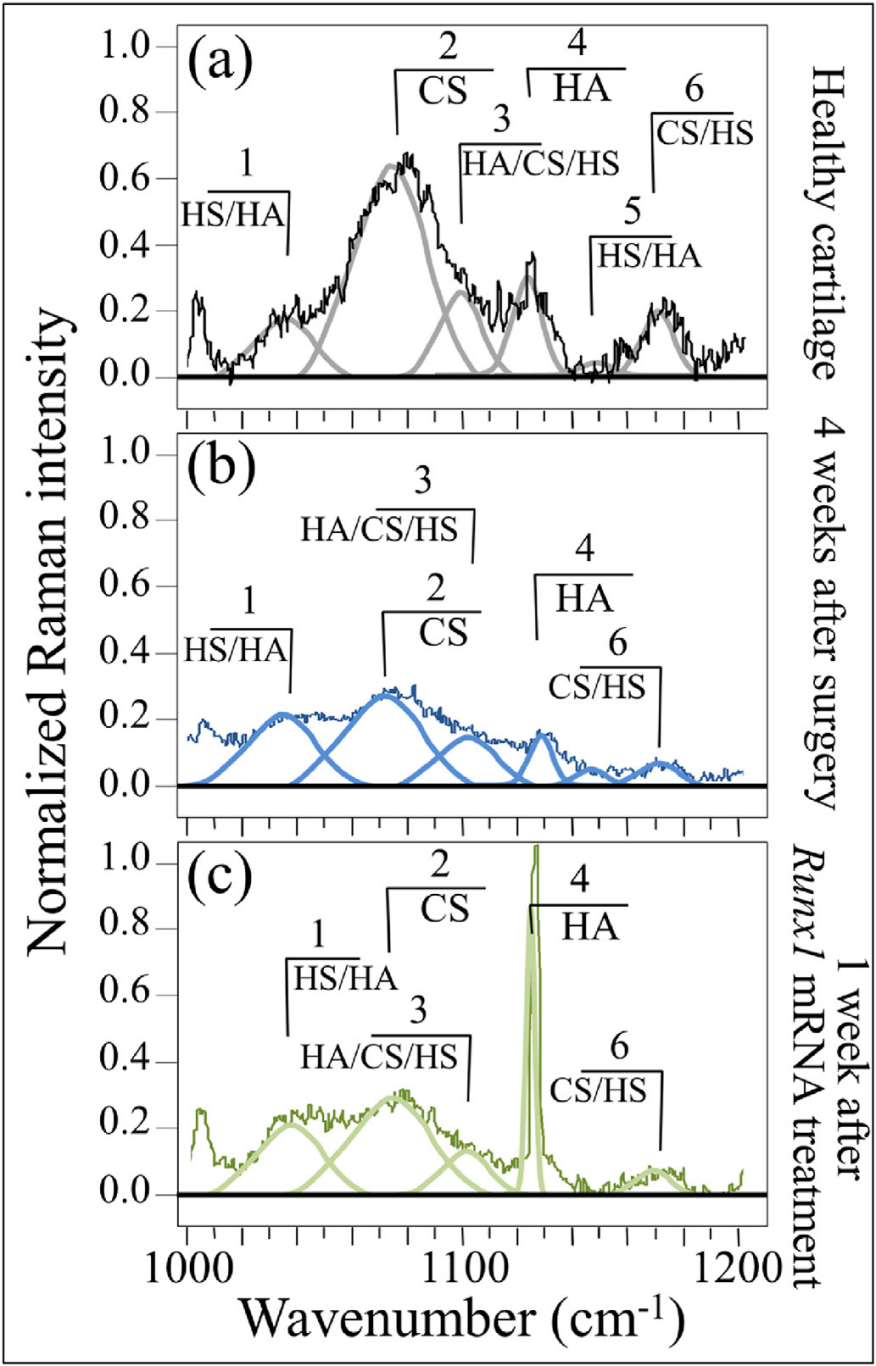
Raman analysis in the spectral zone at 1000–1200 ​cm^−1^: (a) control healthy tibia cartilage, (b) diseased cartilage after 4 weeks since MCL and MM transection, and (c) regenerated cartilage after 4 weeks after MCL and MM transection and one successive week after *Runx1* mRNA administration. Spectral frequencies are given in Table 2 and assignments to specific molecules are given in inset.

Zone II in the spectral interval 1000–1200 ​cm^−1^ is shown in Fig. 6(a), (b), and (c). A strong preponderance of a sharp signal from HA (1123 ​cm^−1^) above that of CS (1065 ​cm^−1^), could clearly be seen in the cartilage treated with *Runx1* mRNA (cf. Fig. 6(c)–. 6(a) and (b)). Conversely, the relative intensity of the band at 1165 ​cm^−1^ (exocyclic –C–O–C– inter-molecule groups) after *Runx1* mRNA treatment was clearly lower than that of healthy cartilage (cf. Fig. 6(a) and (c)). Average Raman spectra collected in this zone on 5 samples for each cartilage condition will be discussed in the forthcoming Section 4.2.

## 4 Discussion

### 4.1 Collagen secondary structures in healthy and degenerated cartilage

The present Raman analyses allowed us to detect fingerprints of proteins' secondary structure in collagen and glycosaminoglycans fractions in the extracellular matrix, thus providing a framework for assessing pathological changes in the cartilage structure. Complementary to histological analyses and optical microscopy, Raman analyses were shown to directly correlate to commonly used standards of cartilage quality [27]. Upon applying these notions to chemical and structural assessments of cartilage before and after regeneration, we have used here Raman spectroscopic data to examine cartilage in healthy, diseased, and regenerated states. As a first attempt, the present Raman approach exploited a comparison between specific bands belonging to the Amide III vibrations in cartilage tissue, as representative of proteins’ secondary structures in collagen [46]. The band located at lower wavenumbers with maximum at ∼1240 ​cm^−1^ is indicative of the presence of random coil, while the band at a higher wavenumber with maximum at ∼1270 ​cm^−1^ represents the α-helix secondary structure. Accordingly, a high relative Raman intensity of the former to the latter band (i.e., the Amide III ratio, *I*_1240_/*I*_1270_) represents a fingerprint of a highly disordered collagen structure, which in turn is a straightforward evidence for the presence of defective collagen in a diseased cartilage tissue. It is known that the α-helix structure undergoes a sharp transition toward the random coil state upon increasing temperature [47]. Accordingly, the most obvious interpretation of the observed increase in Amide III ratio in diseased cartilage is that, after the surgery, the α-helices→random coil transition was triggered by a frictional increase in local temperature.

The most important aspect of these findings is that Raman analyses could detect the earliest stage of osteoarthritic changes at the molecular level. While the Amide III ratio, *I*_1240_/*I*_1270_, showed time-dependent increase from 0.77 to 1.45 for 4 weeks, there were almost no changes in radiological appearance or histologic analysis. Indeed, similar variations could be observed from the intensity ratio of the Amide I band located at 1683 ​cm^−1^ on the one at 1660 ​cm^−1^, *I*_1683_/*I*_1660_ (cf. Fig. S4 in supplementary material), representative of the fractional ratio between disordered and ordered structures in peptides and proteins [48]. Considering the fact that the cartilage degradation become visible around 8 weeks after the surgery of MCL and MM transection [20,30], it is reasonable to say that the molecular changes in the collagen structure is responsible for the following cartilage degeneration. It is also consistent to the study analyzing supramolecular organization of collagen fibrils in human OA cartilage using scanning electron microscopy (SEM), although direct comparison would be difficult because the sample preparation was entirely different from this study [49].

Note that, in a previous study using human knee cartilage after explantation, the variations of Raman Amide III ratio, *I*_1240_/*I*_1270_, were distributed from 0.25 to 0.77 [27]. The values were correlated with the severity of the cartilage degeneration categorized by Collins scale in human knee cartilage, from grade 0 (histologically normal cartilage) through grade IV (almost complete loss of cartilage) [44], corresponded to Raman Amide III ratios, *I*_1240_/*I*_1270_ ​= ​0.25–0.28, 0.29–035, 0.50–0.65, 0.7–0.8, and >0.8, respectively [27]. An important difference between the previous study of human knee cartilage and the present assessment of mice knee cartilage was the value of the *I*_1240_/*I*_1270_ ratio recorded in healthy cartilage, which was significantly higher in mice as compared to humans (∼0.77 vs. ∼0.28). Such a marked difference could be due to either an inherent structural difference of the protein secondary structure between mice and human collagen or to a different degree of alignment of the collagen fibers, or both. In addition, the samples of human knee cartilage were obtained from elderly, which should be different from those of around 12-week-old mice. Nevertheless, the trend toward increased ratios, *I*_1240_/*I*_1270_, with increasing disease severity was basically the same for both species, strongly suggesting that the increased presence of disordered collagen structure would affect the progress of cartilage degeneration.

**Fig. 7:**
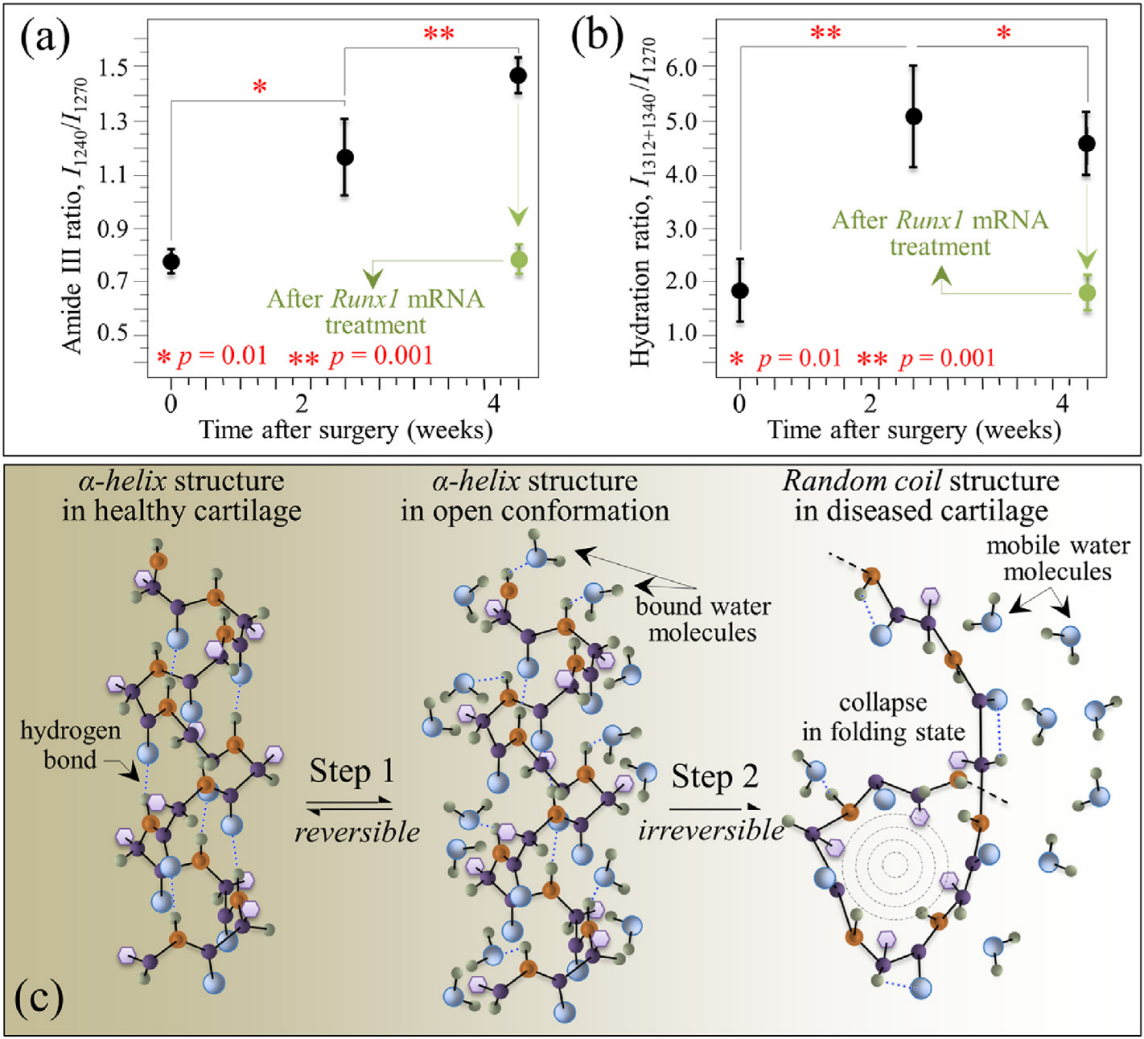
(a) Amide III ratio, *I*_1240_/*I*_1270_, and (b) hydration ratio, *I*_1312+1340_/*I*_1270_, as functions of time after MCL and MM transection (standard deviations and details of statistical validations in inset); the green plots give the value of the two ratios one-week after *Runx1* mRNA administration; in (c), schematic draft of the two-step proposed for disease development: Step 1 → gradual depletion of the internal α-helix hydrogen bonds with formation of external hydrogen bonds to water molecules and concurrent “loosening” toward a more open structure; and, Step 2 → α-helix structure irreversibly collapsing into a random coil configuration.

As indicated previously, an increased friction is thought to be responsible for the initial compositional changes in the cartilage. It is reported that the coefficient of friction in the joint increases significantly with OA progression, particularly in medial zones, due to a decrease in concentration of lubricin, namely the hyaluronate-binding protein found both in synovial fluid and at the cartilage surface [50]. Inspections of mice knee cartilage after 2 and 4 weeks after the surgery recorded Amide III ratios, *I*_1240_/*I*_1270_ ​= ​1.15 and 1.45, respectively (namely, higher than the value in healthy cartilage by ∼49 and 88%, respectively; cf. Fig. 7(a)). Data showed statistical significance, indicating that the disease underwent a relatively fast progression after the surgery. As seen in the radiographs and histological sections in Fig. 1, the cartilage thickness did not collapse even after 4 weeks, although an the advanced state of damage was reached after this period, suggesting that the layer of collagen fibers aligned parallel to the sliding direction nearby the surface had presumably been significantly worn [[51], [52], [53]]. The comparison between the Amide III ratios measured *ex vivo* as a function of time after the surgery shown in Fig. 7(a), which includes data both from before and after cartilage regeneration (discussed later), links the Raman data to the disease severity of OA and further substantiates the physiological meaning of the Raman Amide III ratio.

Another important feature in this region was the increase of signals at ∼1312 and ∼1340 ​cm^−1^, which both belonged to α-helix but in its hydrated state [39,[54], [55], [56]]. In literature studies on this subject, the nature of the hydrated and hydrophobic variants of α-helix could be identified with a new and more open conformation, which leaves the hydrogen-bonding network intact but changes the C–O^…^N angle in the amide plane to allow carbonyls being displayed away from the helix axis into the solution and hydrogens bonding with environmental water molecules. The present Raman data point at a substantial development of water-bound α-helix structure in diseased cartilage after 2 weeks since the surgery (cf. intensification of both signals at ∼1312 and ∼1340 ​cm^−1^ in Fig. 3(b) as compared with Fig. 3(a)). Water indeed plays a major role in controlling both structure and dynamics of macromolecules in healthy and osteoarthritic cartilage [57]. Experiments on knees of mature dogs with natural or surgically induced OA, demonstrated that the overall cartilage thickness of both kinds of diseased joint was more hydrated than the control cartilage [58]. However, hydration loss is reported to occur at later stages of disease and in the tangential layer of cartilage concurrently with a hydration increase in deeper layers [59]. The Raman results of the present study are in line with above notions, as proved by the higher *I*_1240_/*I*_1270_ ratios and more significant hydration (i.e., higher ratios *I*_1312_/*I*_1270_ and *I*_1340_/*I*_1270_) 2 weeks after surgery (cf. Fig. 3). The phenomenon of α-helix hydration was the most pronounced at this early stage of the disease and tended to reduce toward 4 weeks. This suggests the occurrence of a physiological path toward cartilage degradation by frictional heating as disease progresses. Since the main amount of energy lost in friction is in the form of heat, a local temperature increase is expected to strongly affect the molecular structure of cartilage. Paschek et al. [60] showed that, upon thermal unfolding of cartilage peptides, the increase in solvent hydrogen bonding fails to compensate for the loss in internal (helical) hydrogen bonds. Those researchers also showed vibrational data in line with our Raman analyses. Unlike the band at 1312 ​cm^−1^, the intensity of the Raman signal at ∼1340 ​cm^−1^ in healthy cartilage was widely scattered and depended on individual samples. For this reason, we adopted the cumulative intensity of the two bands with respect to the α-helix band at 1270 ​cm^−1^ as representative of the degree of cartilage hydration and plotted the parameter, *I*_1312+1340_/*I*_1270_, as a function of time after the surgery, as shown in Fig. 7(b). Unlike the time dependence of the Amide III ratio (cf. Fig. 7(a)), the hydration ratio, *I*_1312+1340_/*I*_1270_, showed a maximum at 2 weeks after the surgery. This trend suggests a two-step development of the disease with a gradual depletion of the internal hydrogen bonds in the α-helix structure to create a more open α-helix conformation rich in external water-bounds hydrogen bonds (i.e., the Step 1 state of cartilage observed at 2 weeks after the surgery). This loosening process of the α-helix structure is likely a reversible one; but, as it proceeds further, it may reach a stage in which the α-helix structure irreversibly collapses by folding into a random coil (i.e., the Step 2 state of cartilage observed at 4 weeks since surgery). In this latter configuration, water molecules return to their mobile state and, thus, the enhancement in the Raman signals at 1312 and 1340 ​cm^−1^ is lost, while the fraction of random coil continues to increase and so the *I*_1240_/*I*_1270_ ratio. This two-step process, which is graphically shown in Fig. 7(c), allows us to explains the different trends recorded for Amide III and hydration ratios as shown in Fig. 7(a) and (b). Without elaborating further on the difference in absolute values of the *I*_1240_/*I*_1270_ ratio between mice and human knee healthy cartilage, the present study confirms that the Raman Amide III probe contained clear fingerprints that could differentiate normal and pathological cartilage, given its high sensitivity to subtle molecular and structural changes.

### 4.2 Glycosaminoglycans variations in healthy and degenerated cartilage

**Fig. 8:**
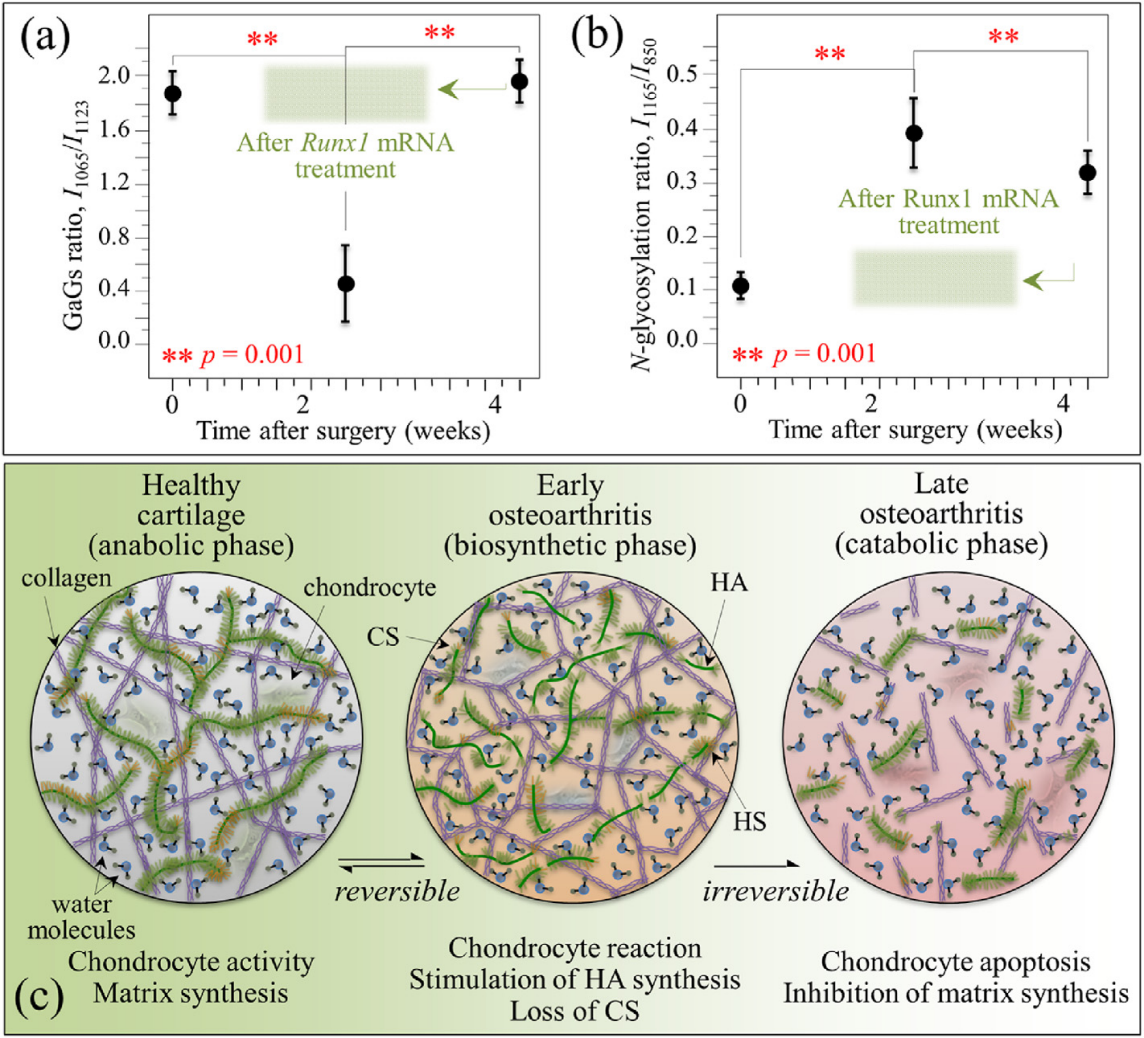
(a) Glycosaminoglycan (GaGs) ratio, *I*_1065_/*I*_1123_, and (b) N-glycosylation ratio, *I*_1165_/*I*_850_, as functions of time after MCL and MM transection (details of statistical validations in inset); the green shadowed areas give the value of the two ratios two-weeks after *Runx1* mRNA administration; in (c), schematic draft of two successive steps in disease development from healthy cartilage: an initial step (biosynthetic phase) in which the chondrocytes yet attempt repairing the diseased extracellular matrix with promoting HA synthesis, and a successive step (degradative phase) in which chondrocytes start producing enzymes that inhibit matrix synthesis and undergo apoptosis. Note that the shown draft only represents what is, in the authors' opinion, the most plausible hypothesis, for explaining the obtained experimental data.

As an additional finding of our present study, we located spectroscopic variations in a Raman region dominated by signals of glycosaminoglycans, which are main constituents of extracellular matrix in cartilage. In comparing diseased cartilage to healthy controls, significant alterations in Raman intensity were noticed for the main peaks of chondroitin sulfate and hyaluronic acid (CS and HA at 1065 and 1123 ​cm^−1^, respectively) [61]. Monitoring the variations of the glycosaminoglycans ratio, *I*_1065_/*I*_1123_, in presence or absence of disease revealed a substantial reduction in CS and a clear increase in HA fractions upon development of OA at 2 weeks since the surgery (Fig. 8(a)). However, the trend for the intensity ratio was restored after 4 weeks since disease development, although the overall Raman intensity of the glycosaminoglycan region was reduced to about half that of healthy cartilage (Fig. 4(c)). The trend observed for the *I*_1065_/*I*_1123_ ratio was exactly the inverse of that of the hydration ratio given in Fig. 7(b), thus showing that significant “loosening” of the α-helix molecular structure by bound water in the collagen fibril structure coincided with a substantial increase in HA above CS in the extracellular matrix.

The finding of a specific loss in CS for osteoarthritic cartilage is in line with a number of previous studies by other authors on both knee and hip human joints [[62], [63], [64]]. It was reported that the loss of negatively charged CS molecules, which are highly hydrophilic and imbibe water, negatively impacts cartilage resilience. On the other hand, our finding of a relatively higher fraction of HA in osteoarthritic cartilage with respect to healthy tissue agrees with a study by Ryu et al. [65]. Note also that Lim et al. [66] reported exactly the same trend observed here (i.e. CS decrease/HA increase) in their study of Raman bands associated with glycosaminoglycans in disrupted porcine cartilage. Considering the fact that HA content is much higher than that of CS in the joint fluid [67,68], it is reasonable to assume that HA molecules were predominantly transitioned into the “loosened” cartilage matrix (Fig. 7), resulting in the increased *I*_1065_/*I*_1123_ ratio at 2 weeks after surgery. However, along with the progress of cartilage damage, both HA and CS were constantly being lost from the cartilage matrix, as represented by the decrease in the overall Raman intensity of the glycosaminoglycan region 4 weeks after the surgery (Fig. 4(c)). From a purely phenomenological viewpoint, the *I*_1065_/*I*_1123_ ratio could be taken as a fingerprint for transition phenomena involving HA variations during the development of the disease and its successive healing.

The increased intensity of the band from exocyclic –C–O–C– bonds (at 1165 ​cm^−1^) after 2 weeks since disease development represents an additional vibrational fingerprint of molecular structure alteration during osteoarthrosis development. The trend in time-lapse of the normalized intensity of this spectroscopic band is given in Fig. 8(b). Normalization was made with respect the C–C stretching signal in protein backbone at ∼850 ​cm^−1^ and the ratio *I*_1165_/*I*_850_ is taken as a parameter representing the level of glycosylation of high-mannose type *N*-glycans in the structure of diseased cartilage. As seen from comparing Fig. 7, Fig. 8, the N-glycosylation ratio follows the same trend of the hydration ratio, showing a maximum at around 2 weeks after the surgery. These results demonstrate that the structure of *N*-glycans was altered upon progressing cartilage OA. N-glycosylation is the result of an enzyme-directed site-specific reaction in which a carbohydrate (glycosyl donor) is attached to a hydroxyl or other functional group of another molecule (glycosyl acceptor; e.g., proteins). Specifically for cartilage, exocyclic oxygen-bridge bonds (–C–O–C–) are present in the long unbranched chains of glycosaminoglycans, which all consist of repeating disaccharide units, and in proteoglycans that are heavily glycosylated proteins. At the present level of investigation, it is not possible to unequivocally assign the transitory increase in 1165 ​cm^−1^ signal observed 2 weeks after the surgery to a specific exocyclic (–C–O–C–)-bond-containing molecule. However, we can hypothesize that the concurrent reduction in the OSO_3_^−^ stretching signal at 1065 ​cm^−1^, which is a fingerprint for CS, rules out a main CS contribution to the 1165 ​cm^−1^ signal. Note that the trend observed in Fig. 8(b) confirms the link between the structures of high-mannose type *N*-glycans and the early processes of human articular cartilage degradation, during which *N*-glycogene influences the regulation of MMP-13 and ADAMTS-5 expression in mouse chondrocytes in response to interleukin-1α (IL-1α) stimulation [45]. Our spectroscopic findings thus confirm the hypothesis that high-mannose-type *N*-glycans are key molecules in OA pathogenesis.

The present Raman data might support the concept that the etiology of OA is the result of complex enzymatic interactions and catabolic-anabolic balance between extracellular matrix and chondrocytes [69]. We also discussed the hypothesis of frictional heating degrading cartilage, a factor that could also be contributing to OA progression. Future work will be required to support or disprove each of the above hypotheses for the OA progression seen in the mice used in this study. However, the Raman data showed that the development of the disease proceeds through two successive steps. In the initial step, the so-called biosynthetic phase, the chondrocytes yet attempt repairing the diseased extracellular matrix with promoting HA synthesis, but they are unable to replace the degraded and folded random coil-structured proteins in collagen with new α–helix ones. However, such biosynthetic anabolic activity is eventually overwhelmed by degradative catabolic activities in which chondrocytes start producing enzymes that inhibit matrix synthesis [70]. Beyond this point, the degeneration of the tissue becomes irreversible. Similar to the interpretation given in Fig. 7(c) for the secondary structure of collagen, we shall interpret the time-lapse Raman data in Fig. 8(a) and (b) as representing the succession of biosynthetic (anabolic) and degradative (catabolic) activities by chondrocytes. This concept is schematically depicted in Fig. 8(c). Note that the draft in Fig. 8(c) represents what, in the authors’ opinion, is the most plausible hypothesis to explain the obtained experimental data.

These results suggest that the Raman feature could be assumed as a fingerprint for cartilage degradation at its early stage since it could be clearly detected before any significant structural disruptions could be found by histology or micro-CT images.

### 4.3 Disease-modifying effects upon Runx1 mRNA administration

The *Runx1* mRNA approach to OA therapy belongs to the category of healing strategies that attempt to modify the structural changes caused by OA through restoring the cartilage original structure [16]. Note, however, that as far as disease-modifying OA drugs are concerned, none of the so far proposed strategies has yet reached medical use [71]. Building upon new knowledge at the molecular level of cartilage degradation in OA pathophysiology [72], the intracellular manipulative path of gene transcription to OA therapy has nowadays reached a realistic level of feasibility. In this context, genetic codes that induce expression *in vivo* of therapeutic transcription factors within target cells perhaps represent the most promising approach to cartilage regeneration. For this purpose, both viral and non-viral vectors have been proposed, although safety concerns and low transfection efficiency have been the main concerns for the former and the latter types of vector, respectively [73]. Both safety and efficiency shortcomings can be overcome by adopting a direct delivery of mRNA into cells, as demonstrated in previous *in vivo* experiments using our original mRNA carrier, polyplex nanomicelles, which allowed for mRNA introduction into the chondrocytes in the cartilage matrix [16]. As an additional advantage, this approach also allowed preventing inflammatory responses (arising from unfavorable immunogenicity of mRNA) and degradation of mRNA (due to highly active RNases in the extracellular space) [22,74].

In the present study, we applied highly spectrally resolved Raman spectroscopy to substantiate at the molecular level the effectiveness of direct *in situ* mRNA delivery of the cartilage-anabolic transcription factor Runx1. A comparison among Raman data collected *ex vivo* on healthy, degenerated, and *Runx1* mRNA-regenerated cartilage from a mouse model of osteoarthritic knee revealed a positive disease-modifying effect, which allowed recovering the original protein secondary structure in collagen fibers, while also restoring the physiological levels of hydration and N-glycosylation in the extracellular matrix. Two main proofs could be given for a restored activity of chondrocytes in diseased cartilage, as follows: (i) recovery of an ordered α-helix structure in the structure of collagen fibrils to replace the disordered random coil structure in diseased cartilage; and, (ii) substantial enhancement of the hyaluronic acid content in the extracellular matrix. Note that both these effects, quantitatively displayed in Fig. 7(a)/(b) and 8(a)/(b), are not possible without a re-established anabolic activity of the chondrocytes. Although subtle orientation change in collagen molecules due to diseases or dehydration might also result in a change in the ratio of *I*_1240_/*I*_1270_ doublet, such damages are not expected to involve a translation from the helix structure to random coil. The present spectroscopic proofs represent the first verification at the molecular scale of the therapeutic effect of the *in vivo Runx1* mRNA approach. Moreover, the Raman characterizations added important information to previous phenomenological findings of therapeutic effects.

As mentioned above, the finding of a recovery of the original secondary structure of cartilage proteins is an important achievement because it proves the occurrence of a restored protein turnover, which is in turn indicative of tissue anabolism and, thus, of a re-established tissue repair capacity associated with the delivery of *Runx1* mRNA. The rationale for this assertion was indeed encrypted in the present Raman data. Four weeks after the surgery, the highly ordered structure of collagen in knee cartilage was irreversibly disrupted as a consequence of friction-related temperature increase. The α-helix structure collapsed into the random coil state, a transition that does not allow spontaneous refolding of the molecule into the ordered α-helix state [37]. Observation of a restored capacity of cartilage regeneration upon *Runx1* mRNA treatment is in line with recent findings by Hsueh et al. [75] according to which an innate repair capacity for postnatal cartilage persists in human adults although it needs to be triggered *ex novo* to reach appreciable joint-healing effects. The full extent to which protein structures returned to their native conditions proved that they have almost completely been exchanged through synthesis by chondrocytes within 2 weeks since the *Runx1* mRNA administration, because no restoration is possible for proteins subjected to heating or to any type of chemically induced unfolding [76].

In the context of extracellular matrix degeneration, an altered balance in glycosaminoglycan fractions is often associated with an increase in tissue hydration [77]. Therefore, the concurrent observations of increased hydration and increased amount of HA in early diseased cartilage are usually consistent to each other (cf. Fig. 7, Fig. 8). In the present study, a further proof of the trend toward cartilage restoration was the high level of HA established upon the *Runx1* mRNA treatment (Fig. 6(c)). While this observation gives a clear hint of a trend toward disease healing at the molecular scale, HA enhancement in the regenerated tissue was not accompanied by tissue hydration (cf. Fig. 7(b)). We interpret the lack of a spectroscopic hydration fingerprint in Runx1 *mRNA*-regenerated cartilage as a direct consequence of establishing a highly ordered α-helix structure by re-activated chondrocytes.

Previous studies [20,78] indicated that Runx1 could promote differentiation of chondrocytes, and thus restore the cartilage anabolic function. Since Runx1 suppresses the progression of OA by mainly activating the remaining chondrocytes, it is strongly suggested that the therapeutic effect would be most expected at the early stage of the disease when the articular chondrocyte population to which mRNA could be delivered is relatively large. Based on comparison of disordered collagen structure between mice and human knee cartilage, as discussed in 4.1, the disease severity at 4 weeks after the surgery, when *Runx1* mRNA was administered in this study, may not be the earliest stage of the disease. Nevertheless, a considerable accomplishment of high HA concentrations in extracellular matrix (and of ordered α-helix structure in collagen fibers) after *Runx1* mRNA treatment should be regarded as a clear proof for the re-establishment of a largely compromised chondrocyte activity through receptors, enzymes, and metabolic pathways. This is in line with previous findings of augmented expressions of cartilage-anabolic markers and a chondrocyte proliferation marker in the chondrocytes after *Runx1* mRNA treatment [20].

**Fig. 9:**
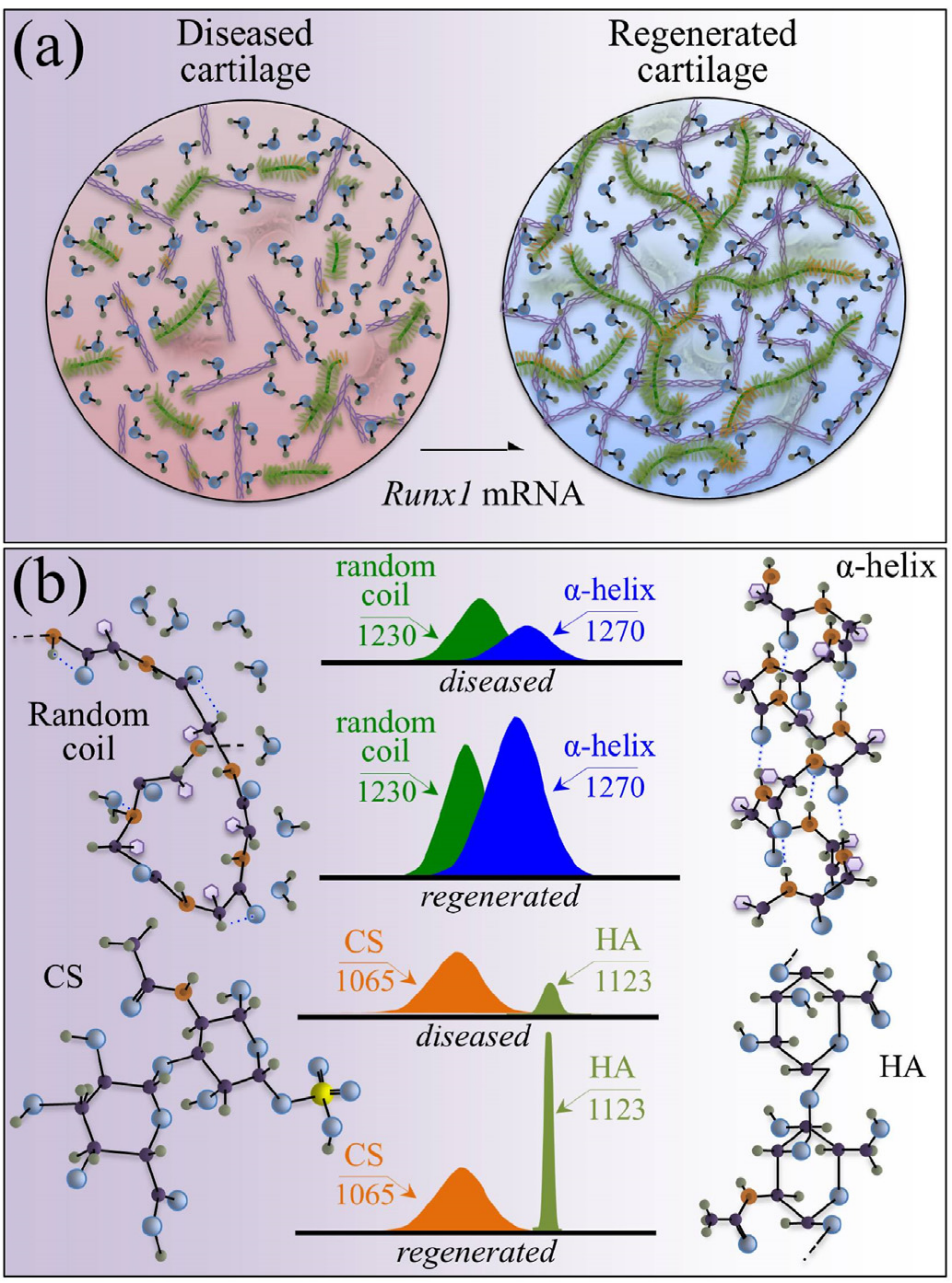
(a) Putative therapeutic action of *Runx1* mRNA in terms of chondrocyte activation; and, (b) the spectroscopic fingerprints of cartilage regeneration at the molecular scale.

As this study is the first trial to analyze the earliest changes in the cartilageit, there are still many details that need to be clear. Waiting for further clinical and analytical verifications of the direct actions of Runx1 on osteophyte formation and chondrocyte hypertrophy, we demonstrated its positive role in chondrocyte activation and confirmed its therapeutic effects at the molecular level. The therapeutic action of the *Runx1* mRNA treatment in terms of chondrocyte activation and the spectroscopic proofs for it are summarized and schematically drawn in Fig. 9(a) and (b), respectively.

## 5 Conclusion

In summary, the present Raman characterizations gave a consistent picture of various degradation phenomena that take place in osteoarthritic cartilage. They also demonstrated the possibility of consistently matching information of proteins’ secondary structure (from Amide III vibrations), level of hydration (from hydrated *N*–H bending and C–N stretching), fractions of different glycosaminoglycans (from OSO_3_^−^ and C–H/C–OH vibrations), and levels of N-glycosylation (from exocyclic –C–O–C– vibrations), as obtained from selected Raman vibrational fingerprints in a comprehensive assessment of cartilage diagnostics.

In addition, we have taken a further step in clarifying the therapeutic effects of the *Runx1* mRNA procedure in the context of cartilage regeneration by means of Raman spectroscopy. Raman spectra were systematically collected *ex vivo* from selected zones of in mice's knee before/after the surgery of MCL and MM transection, and after the mRNA administration. The secondary structure of cartilage proteins (Amide III ratio, *I*_1240_/*I*_1270_), the level of cartilage hydration (*I*_1312+1340_/*I*_1270_ ratio), the variations in glycosaminoglycan fractions (*I*_1063_/*I*_1126_ ratio), and the extent of glycosylation in *N*-glycans (ratio *I*_1165_/*I*_850_) were targeted and probed. The spectral changes of Amide III and glycosaminoglycans ratios substantiated a process of molecular-scale repair from pathological abnormality upon *Runx1* mRNA administration. Since the visible optics used by Raman instruments is compatible with fiber-optic probes used in clinical arthroscopes, the Raman parameters proposed in this study could be used in the future for monitoring OA *in vivo* and assessing in real time cartilage regeneration processes at the molecular scale.

## Credit author statement

Giuseppe Pezzotti: conception, data interpretation, have drafted the work and revised it. Wenliang Zhu: acquisition and data interpretation. Yuki Terai: data acquisition. Elia Marin: data interpretation. Francesco Boschetto: data interpretation. Komei Kawamoto: data acquisition. Keiji Itaka: conception, data acquisition, data interpretation, and revision.

## Arrive guidelines statement

The authors have read the ARRIVE guidelines, and the manuscript was prepared according to the ARRIVE guidelines.

## Declaration of competing interest

The authors declare that they have no known competing financial interests or personal relationships that could have appeared to influence the work reported in this paper.
