# Supplementary material for: Raman spectroscopic insight into osteoarthritic cartilage regeneration by mRNA therapeutics encoding cartilage-anabolic transcription factor Runx1
Source: Mater Today Bio. 2022 Jan 29;13:100210. doi: 10.1016/j.mtbio.2022.100210 (PMC8913780; doi:10.1016/j.mtbio.2022.100210)
Supplement: Multimedia component 1 [file mmc1.docx]

**Raman spectroscopic insight into osteoarthritic cartilage regeneration by mRNA therapeutics encoding cartilage-anabolic transcription factor Runx1**

Giuseppe Pezzotti, Wenliang Zhu, Yuki Terai, Elia Marin, Francesco Boschetto,

Komei Kawamoto, and Keiji Itaka

**Supplemantary Figures**

Supplementary Figure 1

On-chip capillary electrophoresis of mRNA encoding hRUNX1 ([NM_001754.5]; 1443 bps) using Bioanalyzer Agilent2100 (Agilent, Santa Clara, CA, USA).

Supplementary Figure 2

Micro-CT images of mice knee at 2-, 4-, and 8-week after the surgery.

Supplementary Figure 3

Histological-section images of mouse knee of a different mouse from those shown in Fig. 1a.

Supplementary Figure 4. Raman spectra in the range from 1610 to 1725 cm^-1^ of recorded *ex vivo* on control healthy tibia cartilage, those from knee joints at 2 and 4 weeks after MCL and MM transection, and one successive week after *Runx1* mRNA administration.

**Supplementary Figure 1. On-chip capillary electrophoresis of mRNA encoding *hRUNX1*** **([NM_001754.5]; 1443 bps) using Bioanalyzer Agilent2100 (Agilent, Santa Clara, CA, USA).**

(A)


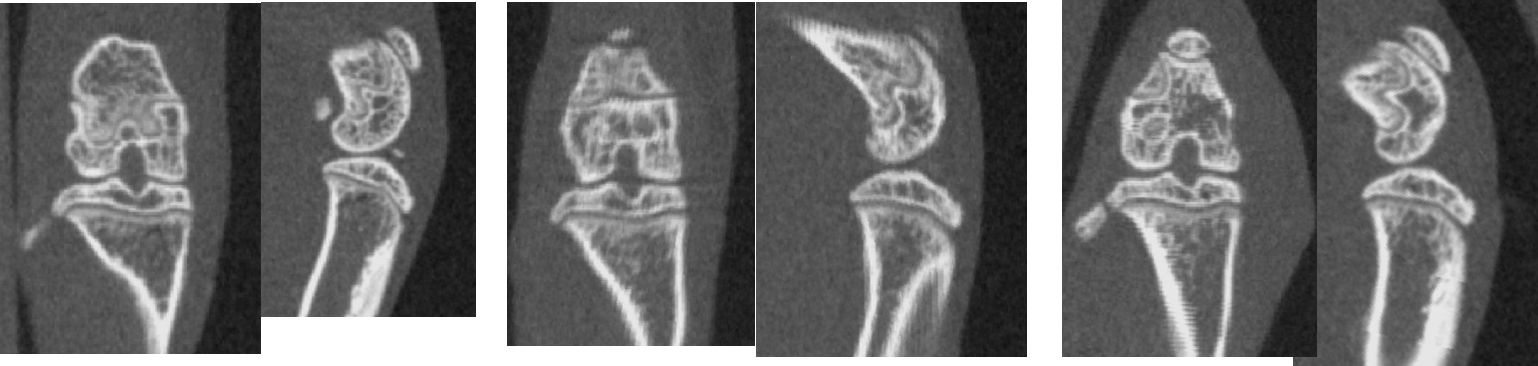


(B)


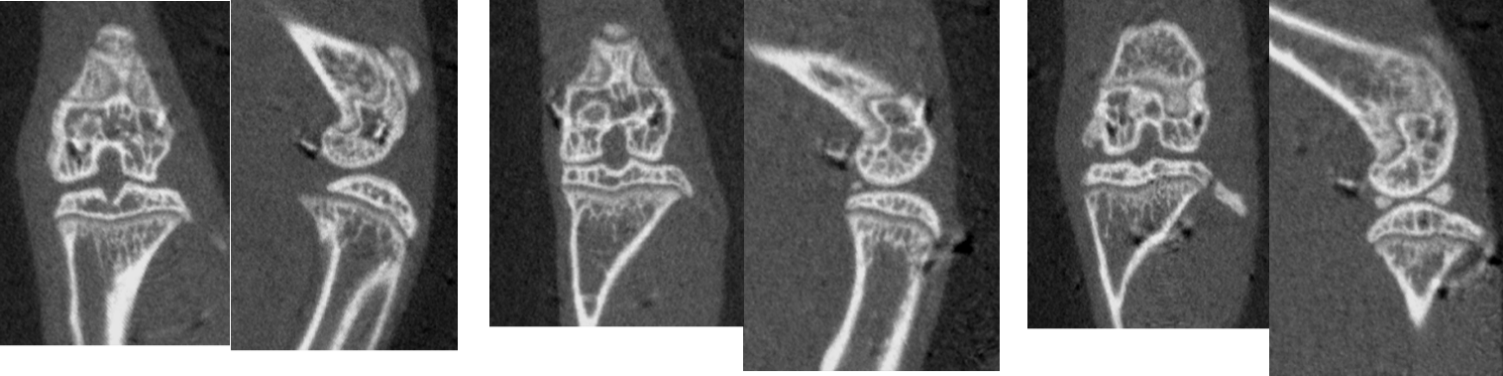


(C)


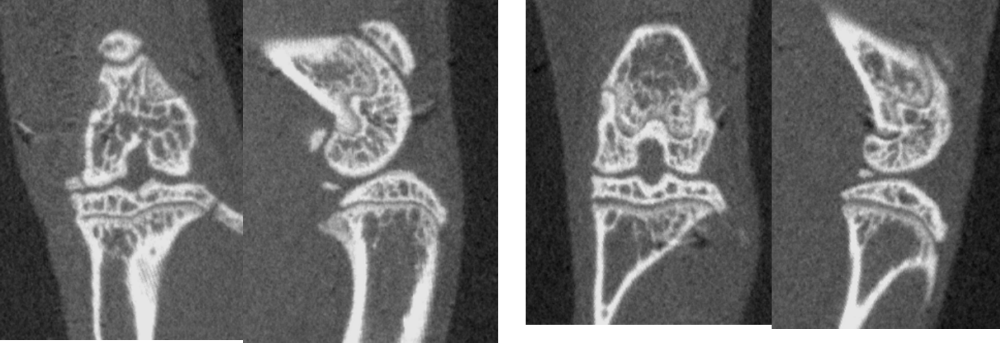


**Supplementary Figure 2. Micro-CT images of mice knee at (A) 2 weeks, (B) 4 weeks, and (C) 8 weeks after MCL and MM transection. There are almost no significant OA changes in the knee joint.**


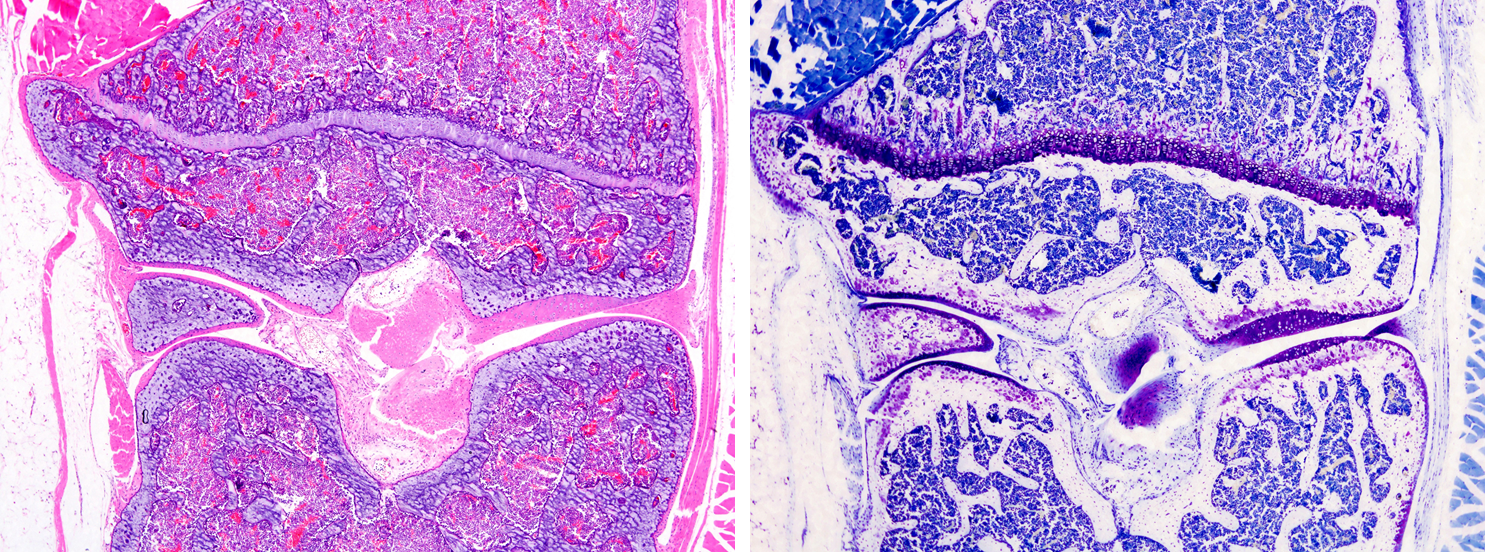


**Supplementary Figure 3. Histological-section images of mouse knee of a different mouse from those shown in Fig. 1a in the main text, obtained at 4 weeks after MCL and MM transection. The sections were stained with hematoxylin and eosin (left) or toluidine blue (right).**


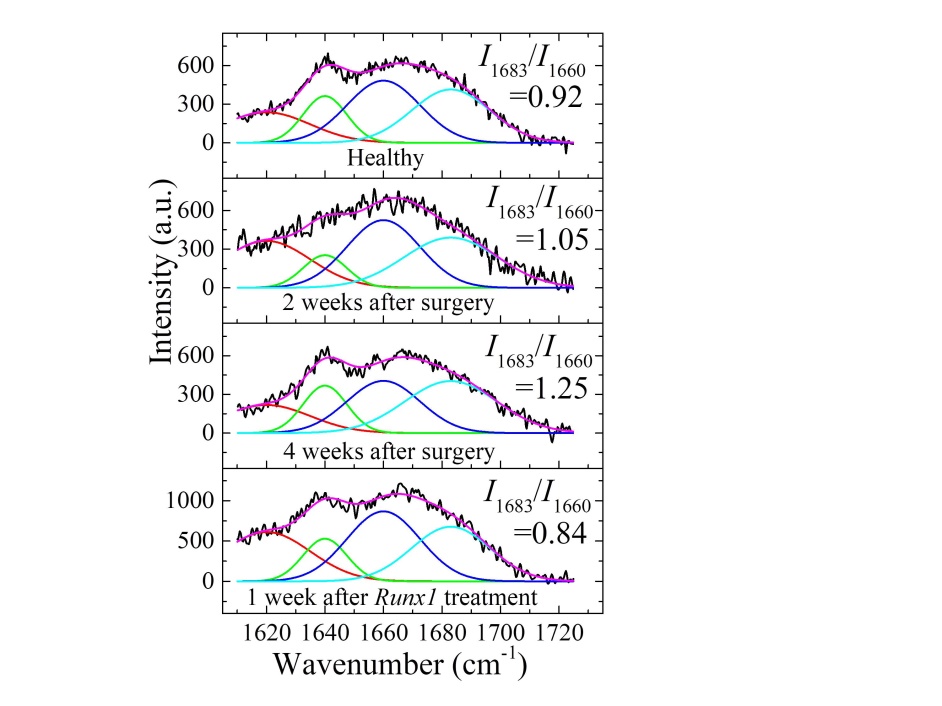


**Supplementary Figure 4. Raman spectra in the range from 1610 to 1725 cm^-1^ of recorded *ex vivo* on control healthy tibia cartilage, those from knee joints at 2 and 4 weeks after MCL and MM transection,** **and one successive week after *Runx1* mRNA administration. The bands are assigned to the vibrational modes of Amide I, while the exact spectral location strongly depends on the secondary structure of proteins, related to both α-helix (1660 cm^-1^) and β-sheet (1640 cm^-1^), as well as to a disordered structure of α-helix (1683 cm^-1^). The intensity ratio of *I*_1683_/*I*_1660_ is representative of the fractional ratio between disordered and ordered structures in peptides and proteins.**
